# TGF-***β*** Signaling Cooperates with AT Motif-Binding Factor-1 for Repression of the ***α***-Fetoprotein Promoter

**DOI:** 10.1155/2014/970346

**Published:** 2014-07-03

**Authors:** Nobuo Sakata, Satoshi Kaneko, Souichi Ikeno, Yutaka Miura, Hidekazu Nakabayashi, Xue-Yuan Dong, Jin-Tang Dong, Taiki Tamaoki, Naoko Nakano, Susumu Itoh

**Affiliations:** ^1^Laboratory of Biochemistry, Showa Pharmaceutical University, 3-3165 Higashi-Tamagawagakuen, Machida, Tokyo 194-8543, Japan; ^2^Department of Molecular Neurobiology, Nagoya City University Graduate School of Medical Sciences, Nagoya 467-8601, Japan; ^3^Department of Medical Management & Information, Hokkaido Information University, Ebetsu, Hokkaido 069-8585, Japan; ^4^Department of Hematology and Medical Oncology and Winship Cancer Institute, Emory University School of Medicine, Atlanta, GA 30322, USA; ^5^Department of Biochemistry and Molecular Biology, University of Calgary, Calgary, AB, Canada T2N 4N1

## Abstract

*α*-Fetoprotein (AFP) is known to be highly produced in fetal liver despite its barely detectable level in normal adult liver. On the other hand, hepatocellular carcinoma often shows high expression of AFP. Thus, AFP seems to be an oncogenic marker. In our present study, we investigated how TGF-*β* signaling cooperates with AT motif-binding factor-1 (ATBF1) to inhibit *AFP* transcription. Indeed, the expression of *AFP* mRNA in HuH-7 cells was negatively regulated by TGF-*β* signaling. To further understand how TGF-*β* suppresses the transcription of the *AFP* gene, we analyzed the activity of the *AFP* promoter in the presence of TGF-*β*. We found that the TGF-*β* signaling and ATBF1 suppressed *AFP* transcription through two ATBF1 binding elements (AT-motifs). Using a heterologous reporter system, both AT-motifs were required for transcriptional repression upon TGF-*β* stimulation. Furthermore, Smads were found to interact with ATBF1 at both its N-terminal and C-terminal regions. Since the N-terminal (ATBF1N) and C-terminal regions of ATBF1 (ATBF1C) lack the ability of DNA binding, both truncated mutants rescued the cooperative inhibitory action by the TGF-*β* signaling and ATBF1 in a dose-dependent manner. Taken together, these findings indicate that TGF-*β* signaling can act in concert with ATBF1 to suppress the activity of the *AFP* promoter through direct interaction of ATBF1 with Smads.

## 1. Introduction

The oncofetal glycoprotein *α*-fetoprotein (AFP) is a major serum protein expressed at high levels in the yolk sac and liver during embryonic development [1, 2]. However, AFP in adult serum is undetectable except in patients who suffer from hepatocellular carcinoma (HCC). Thus, AFP is a useful tumor marker for measuring the malignancy grade of HCC [3]. The human* AFP *gene is activated by hepatocyte nuclear factor-1 (HNF-1), which can bind to an AT-motif in the proximal and/or distal promoter region of* AFP* [4–6].

AT motif-binding factor-1 (ATBF1) encodes a protein comprising multiple zinc fingers and homeodomains [7, 8]. ATBF1 was originally discovered as a negative transcriptional regulator of the human* AFP* gene, which competes with HNF-1 for binding to the AT-motifs [4]. Thus, ATBF1 seems to act as a transcriptional repressor of the* AFP* gene [4, 9, 10]. Besides the* AFP* gene, ATBF1 can also negatively regulate the transcription of the* Myb *gene [11]. When ATBF1 was overexpressed in C2C12 cells, the expression of* Id3* and* cyclin D* increased, whereas that of* MyoD* and* myogenin* did not [12]. Thus, ATBF1 seems to play a key role not only as a negative, but also as a positive transcriptional regulator. Recently,* ATBF1* was suspected to be a candidate tumor suppressor gene because it is frequently mutated or deleted in prostate, breast, and gastric tumors, and its expression is also suppressed in some tumors [13–17].

Transforming growth factor-*β* (TGF-*β*) regulates a great number of cellular responses including proliferation, differentiation, apoptosis, extracellular matrix production, motility, and immunosuppression. TGF-*β* elicits its cellular effects by making a heteromeric complex between TGF-*β* type I (T*β*RI, also termed activin receptor-like kinase 5 [ALK5]) and TGF-*β* type II receptors (T*β*RII). After TGF-*β* binds to T*β*RII, T*β*RI is recruited by T*β*RII to be phosphorylated within its GS domain by the constitutively active T*β*RII kinase. The activated T*β*RI kinase initiates signaling through phosphorylation of specific receptor-regulated Smads (R-Smads, i.e., Smad2 and Smad3). Subsequently, two activated R-Smads form ternary complexes with a common-partner Smad (Co-Smad), Smad4. These complexes can regulate TGF-*β*-responsive genes either directly via their binding to the promoters of these genes or indirectly via their association with a large number of transcription factors, coactivators, and/or corepressors. Of interest, Co-Smad and R-Smads except for Smad2 possess the ability to bind to specific DNA sequences, whereas Smad2 has 30-aa insertion, immediately prior to obvious DNA binding region in its MH1 domain, which prevents Smad2 from binding to DNA [18–20].

TGF-*β* is known to negatively regulate* AFP* expression [21, 22]. Furthermore, TGF-*β*-mediated repression of* AFP* involves recruitment of Smad to p53 together with SnoN and mSin3A in the AFP promoter [22, 23]. Although ATBF1 also contributes to negative regulation of* AFP*, how TGF-*β* signaling cooperates with ATBF1 to inhibit* AFP* transcription remains veiled. In this study, we could confirm that the TGF-*β*/Smad signaling pathway plays an inhibitory role in the* AFP* transcription via the interaction of Smads with ATBF1.

## 2. Materials and Methods

### 2.1. Cell Culture

COS7 cells, monkey kidney fibroblast-like cells, were cultured in Dulbecco's modified Eagle's medium (Nacalai) containing 10% fetal calf serum (FCS; Invitrogen). HepG2 and HuH-7 cells, hepatocellular carcinoma cells, were maintained in Eagle's minimum essential medium (Wako) containing 10% FCS, 1% nonessential amino acids, and 1% sodium pyruvate. All media were supplemented with 100 IU/mL penicillin and 100 *μ*g/mL streptomycin.

### 2.2. Plasmid Constructions

The expression constructs for HA-ATBF1, Myc-ATBF1, HA-ATBF1N, HA-ATBF1M, and HA-ATBF1C have been previously described [24, 25]. The AFP luciferase reporters and Flag-Smad2, Flag-Smad3, and Flag-Smad4 have been documented [6, 26, 27]. Various lengths of the 5′promoter region of human* AFP* were cloned by PCR and inserted into the luciferase gene with either TK (TK.Luc) or CMV minimal promoter (CMV Luc). Mutation in the* AFP* promoter was performed using a KOD-Plus-Mutagenesis kit (TOYOBO). Adenoviral ALK5ca was generously provided by Fujii et al. [28].

### 2.3. Luciferase Reporter Assay

HepG2 cells were seeded at 1.5 × 10^5^ cells/well in a 12-well plate one day before transfection. The cells were transfected with reporter constructs using polyethyleneimine (PEI). After 24 h of transfection, cells were stimulated with 5 ng/mL TGF-*β* for 18 h. In all luciferase assays, *β*-galactosidase activity in cells transfected with 100 ng of pCH110 (GE Healthcare) was measured to normalize the transfection efficiency. The results were the averages of three independent transfections and were repeated at least twice. The representative data were shown. All values represent mean ± S.D. Each *t*-test between two columns was performed: *P < 0.05, **P < 0.01, and ***P < 0.001.

### 2.4. RT-PCR Analysis

Total RNAs from HuH-7 cells were extracted using an RNeasy Plus Mini kit (Qiagen). Reverse transcription was carried out using a High-Capacity RNA to cDNA kit (Applied Biosystems). PCR was performed using GoTaq (Promega) according to the manufacturer's instructions.

### 2.5. Immunoprecipitation and Western Blot Analysis

To detect interactions among the proteins, plasmids were transfected into COS7 cells (5 × 10^5^ cells/6 cm dish) using PEI. Forty hours after the transfection, cells were lysed in 500 *μ*L of TNE buffer (10 mM Tris (pH 7.4), 150 mM NaCl, 1 mM ethylenediamine-*N*′,*N*′,*N*′,*N*′-tetraacetic acid (EDTA), 1% NP-40, 1 mM phenylmethylsulfonyl-l-fluoride (PMSF), 5 *μ*g/mL leupeptin, 100 U/mL aprotinin, 2 mM sodium vanadate, 40 mM NaF, and 20 mM *β*-glycerophosphate). The cell lysates were precleared with protein G-Sepharose beads (GE Healthcare) for 30 min at 4°C and then incubated with anti-Myc or anti-Flag antibody (Sigma) for 2 h at 4°C. The protein complexes were immunoprecipitated by incubation with protein G-Sepharose beads for 30 min at 4°C followed by three washes with TNE buffer. The immunoprecipitated proteins and aliquots of the total cell lysates were boiled for 5 min in sample buffer, separated by SDS-polyacrylamide gel electrophoresis (SDS-PAGE), and transferred to a Hybond-C Extra membrane (GE Healthcare). The membranes were probed with primary antibodies. The primary antibodies were detected with horseradish peroxidase-conjugated secondary antibodies and chemiluminescent substrate (Thermo Scientific). Protein expression in the total cell lysates was evaluated by Western blot analysis.

### 2.6. DNA Affinity Precipitation (DNAP)

COS7 cells were seeded at 1.5 × 10^6^ cells/well in a 10 cm dish one day before transfection. Ten micrograms of indicated expression vectors were transfected into COS7 cells using PEI. Forty hours after the transfection, the cells were lysed in 1 mL of TNE buffer. Then, each cell lysate was divided and mixed for DNAP. The combined cell lysates were precleared with 12 *μ*g/mL poly (dI · dC) and streptavidin agarose (Sigma) for 30 min and then incubated with 24 *μ*M biotinylated (AT-motif)_3_ for 2 h at 4°C. Subsequently, streptavidin agarose was added to the reaction mixture and incubated for 30 min at 4°C. After the precipitates had been washed with TNE buffer three times, the precipitates and aliquots of the total lysates were separated by SDS-PAGE. The proteins were then transferred to the membrane. The membrane was incubated with the indicated primary antibodies. The primary antibodies were detected as described above. The sequences of the biotinylated (AT-motif)_3_ were as follows: 5′-biotinylated CTCGAGGCTGTTAATTATTGGGGCTGTTAATTATTGGGGCTGTTAATTATTGAATTC-3′/3′-GAATTCAATAATTAACAGCCCCAATAATTAACAGCCCCAATAATTAACAGCCTCGAG-5′.

## 3. Results

### 3.1. AFP mRNA Is Inhibited upon TGF-*β* Stimulation

**Figure 1:**
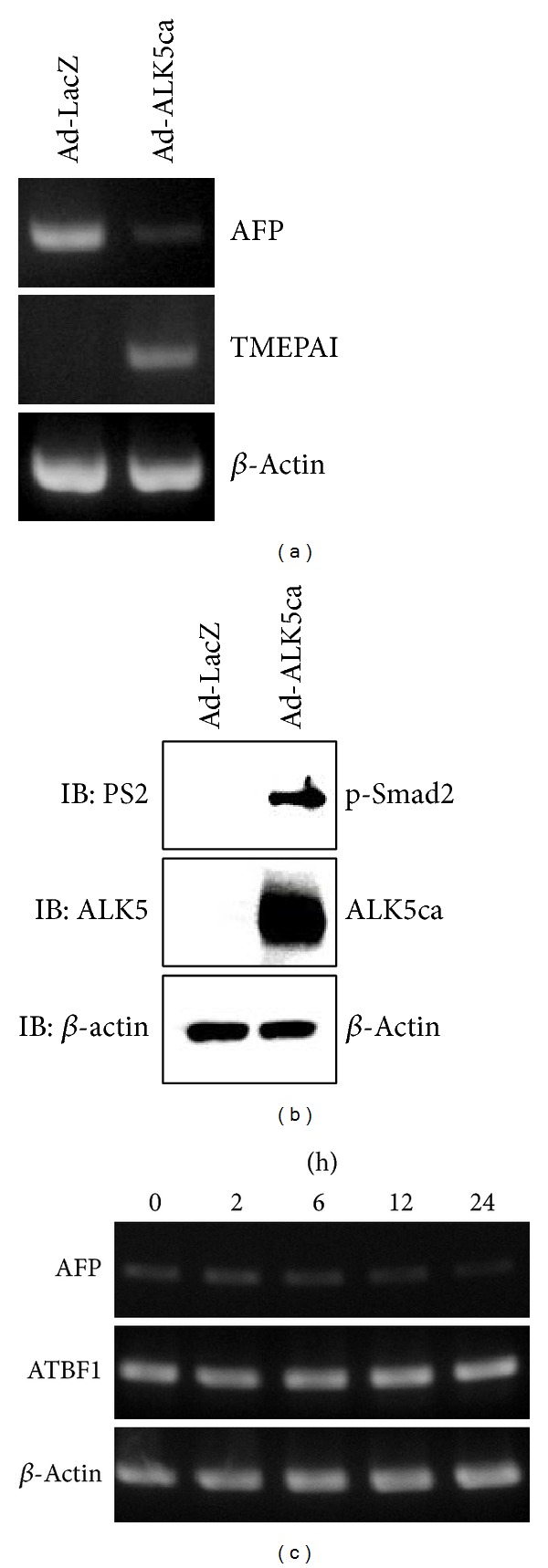
TGF-*β* represses AFP mRNA. (a) AFP mRNA expression was inhibited by ALK5ca in HuH-7 cells. The cells were infected with LacZ (left lane) or ALK5ca-expressing adenoviruses (right lane) at a dose of 250 multiplicity of infection. After 42 hours of infection, total RNA and cell lysates were prepared for RT-PCR analysis (a) and Western blot analysis (IB) (b), respectively. (a) Expression of AFP, TMEPAI, and *β*-actin mRNA was detected by RT-PCR. TMEPAI which is a direct target gene of TGF-*β* signal [29–31] was used as a positive control. (b) For IB, antiphosphorylated Smad2 (PS2), anti-ALK5, and anti-*β*-actin antibodies were used. (c) Expression of AFP mRNA upon TGF-*β* stimulation. HuH-7 cells were treated with 5 ng/mL TGF-*β* for the indicated times. Then, RT-PCR was performed as described in (a). Each PCR condition is described in Table 1.

It has been reported that TGF-*β* contributes to the suppression of the activity of the* AFP* promoter [21, 22]. Indeed, we examined if the expression of AFP mRNA was affected in HuH-7 cells upon ALK5 activation. AFP mRNA was considerably reduced in cells infected with constitutively active ALK5 (ALK5ca) expressing adenovirus, whereas TMEPAI mRNA which has been known to be a direct target gene of TGF-*β*/Smad signal [29–31] was remarkably induced (Figure 1(a)). Furthermore, AFP mRNA slightly decreased with time when HuH-7 cells were stimulated with TGF-*β*. On the other hand, ATBF1 mRNA did not change upon TGF-*β* stimulation (Figure 1(c)).

### 3.2. TGF-*β*/Smad Signaling Inhibits the Activity of the* AFP* Promoter Together with ATBF1

**Figure 2:**
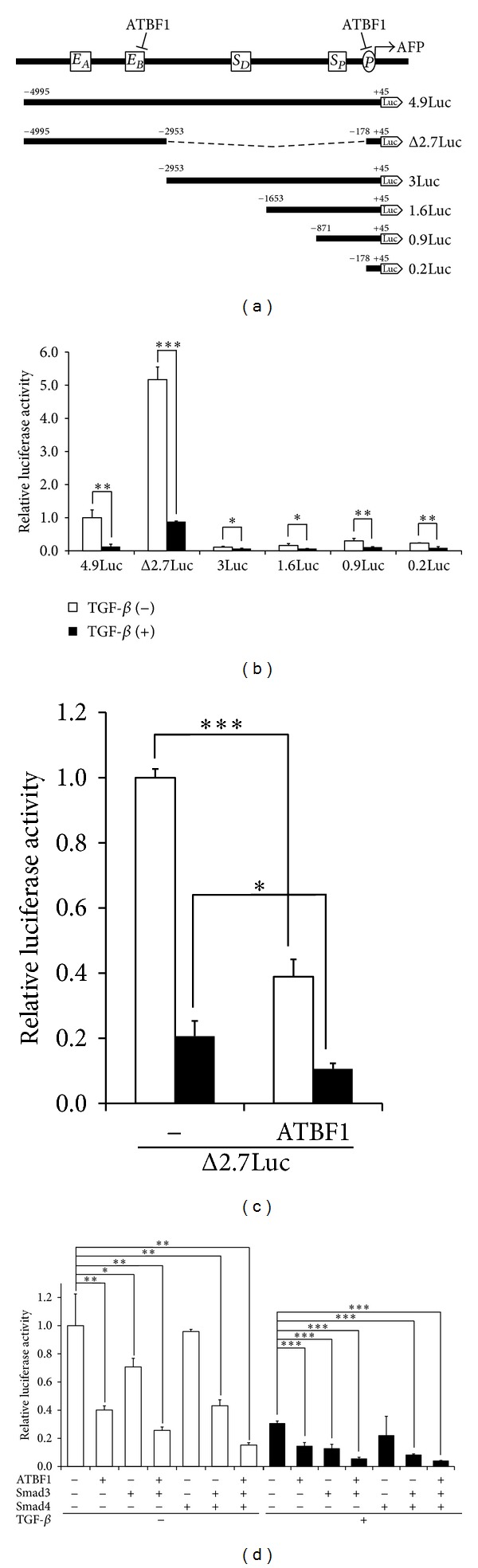
TGF-*β*/Smad signaling inhibits the activity of the* AFP* promoter together with ATBF1. (a) Schematic presentation of deletion mutants of the* AFP* promoter. *E*_*A*_ between −4120 and −3756 and *E*_*B*_ between −3492 and −3300 are enhancer elements. *S*_*D*_ between −1807 and −1725 and *S*_*P*_ between −317 and −300 are silencer elements. P indicates a promoter. (b) Effect of TGF-*β* on the activity of the* AFP *promoter. The examined luciferase reporters (500 ng) were transfected into HepG2 cells. The cells were then stimulated with 5 ng/mL TGF-*β*. (c) Cooperation of ATBF1 with the TGF-*β* signaling for suppression of the activity of the* AFP* promoter. ATBF1 expression plasmid (200 ng) was transfected into HepG2 cells with Δ2.7Luc (500 ng). The cells were then stimulated with 5 ng/mL TGF-*β*. (d) Effect of Smads and ATBF1 on the transcriptional activity of Δ2.7Luc. HepG2 cells were transfected with Smads (100 ng), ATBF1 (200 ng), or their combination together with Δ2.7Luc (500 ng). The cells were then stimulated with 5 ng/mL TGF-*β*.

It has already been demonstrated that enhancer elements (*E*_*A*_, *E*_*B*_), silencer elements (*S*_*D*_, *S*_*P*_), and a promoter (P) are present in the 5′ flanking region of* AFP* gene [5, 32–34]. ATBF1 is known to bind to *E*_*B*_ and P to repress the activity of the* AFP *promoter [4, 6, 26]. To evaluate the involvement of these elements in TGF-*β*-mediated repression of the activity of the* AFP *promoter, each luciferase reporter shown in Figure 2(a) was transfected into HepG2 cells. The cells were then stimulated with TGF-*β*. As shown in Figure 2(b), both 4.9Luc and Δ2.7Luc, which possessed a high basal level of the reporter activity, obviously showed TGF-*β*-mediated repression of the reporter activity. 3Luc, 1.6Luc, and 0.9Luc contain Smad binding element/p53 response element (SBE/p53RE) which has been reported to confer TGF-*β*-mediated repression of the* AFP* promoter [22, 23]. Indeed, these reporter activities were also reduced upon TGF-*β* stimulation in spite of a very low basal activity of their luciferase reporters. Like these three reporters, 0.2Luc, which possesses one AT-motif, could exhibit TGF-*β*-mediated repression of the luciferase activity as well (Figure 2(b)). These results supported the notion that the region between −4995 and −2953 concededly includes* cis*-element(s) for TGF-*β* to suppress the activity of the* AFP *promoter. To test the possibility that ATBF1 can suppress the activity of the* AFP* promoter upon TGF-*β* stimulation, Δ2.7Luc was cotransfected with or without ATBF1. Subsequently, the cells were stimulated with TGF-*β*. As seen in Figure 2(c), ATBF1 could further reduce the luciferase activity that was repressed by TGF-*β*. Since Smad3 and Smad4 play key roles in the canonical TGF-*β* signaling pathway [18, 20], we investigated whether Smad3 and/or Smad4 are implicated in TGF-*β*-mediated repression of the* AFP *promoter together with ATBF1. As shown in Figure 2(d), Smad3 alone or the combination of Smad3 with Smad4 suppressed the reporter activity. Moreover, ATBF1 further diminished the reporter activity that was restrained by both Smad3 and Smad4 upon TGF-*β* stimulation.

### 3.3. Requirement of ATBF1 Binding Sites for Transcriptional Repression of the* AFP* Gene by TGF-*β*

**Figure 3:**
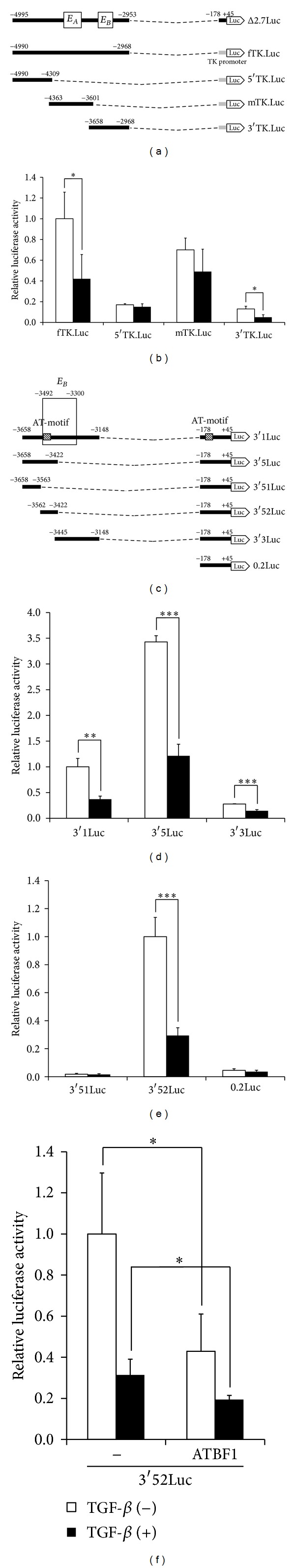
Requirement of a distal AT-motif for TGF-*β*-mediated repression of the activity of the* AFP* promoter. (a) Schematic presentation of TK.Luc conjugated with several fragments of the* AFP *promoter between −4990 and −2968. (b) Effect of TGF-*β* on the activity of TK.Luc reporters conjugated with several truncated* AFP* promoter regions. Each reporter (200 ng) was transfected into HepG2 cells. The cells were then stimulated with 5 ng/mL TGF-*β*. (c) Schematic presentation of 0.2Luc conjugated with several fragments of the* AFP* promoter between −3658 and −3148. (d) Effect of TGF-*β* on the activity of the luciferase reporters described in Figure 3(c). Each reporter (200 ng) was transfected into HepG2 cells. The cells were then stimulated with 5 ng/mL TGF-*β*. (e) Requirement of the fragment between −3562 and −3422 for TGF-*β*-mediated suppression of the* AFP* promoter. HepG2 cells were transfected with the indicated plasmids (200 ng). The cells were then stimulated with 5 ng/mL TGF-*β*. (f) Cooperation of ATBF1 with the TGF-*β* signaling for 3′52Luc activity. HepG2 cells were transfected with either 3.52Luc reporter (200 ng) in the presence or absence of ATBF1 (200 ng). The cells were then stimulated with 5 ng/mL TGF-*β*.

Because the region between −4995 and −2953 is important for the TGF-*β*/Smad signaling to cooperate with ATBF1 for the suppression of the activity of the* AFP *promoter, we speculated that *E*_*B*_, which includes the ATBF1 binding site, is involved in an inhibitory action via the TGF-*β*/Smad signaling pathway. To test this possibility, the region between −4990 and −2968 was divided into three pieces. Then, each fragment was ligated to the luciferase reporter driven by the herpes virus thymidine kinase promoter (TK.Luc) (Figure 3(a)). As shown in Figure 3(b), TGF-*β* suppressed the reporter activity of fTK.Luc. Thus, the region between −4990 and −2968 possesses an inhibitory* cis*-element(s) upon TGF-*β* stimulation. Within this region, the fragment between −3658 and −2968 (termed 3′TK.Luc) revealed a remarkably inhibitory* cis*-element by TGF-*β*, although TGF-*β*-mediated repression of the reporter activity could also be seen in mTK.Luc. Intriguingly, *E*_*B*_ containing the AT-motif is present between −3492 and −3300 (Figure 3(c)). To further narrow down a TGF-*β*-responsive inhibitory* cis*-element(s), we made four luciferase reporters that were linked to the* AFP* minimal promoter (Figure 3(c)). TGF-*β* dramatically suppressed the luciferase activity of 3′1Luc, 3′5Luc, and 3′52Luc although 3′3Luc activity was also inhibited by TGF-*β* to some extent in spite of its low basal luciferase activity, indicating that the region from −3562 to −3422 possesses a TGF-*β*-mediated inhibitory* cis*-element if not more (Figures 3(d) and 3(e)). Thus, an AT-motif and/or its adjacent sequences in *E*_*B*_ were highly suggestive of a TGF-*β*-mediated inhibitory* cis*-element. In addition, ATBF1 could further inhibit the 3′52Luc activity suppressed by TGF-*β* (Figure 3(f)). Therefore, it is possible that the TGF-*β*/Smad pathway interferes via the region between −3562 and −3422.

### 3.4. A Role of the Proximal AT-Motif in the* AFP* Promoter

**Figure 4:**
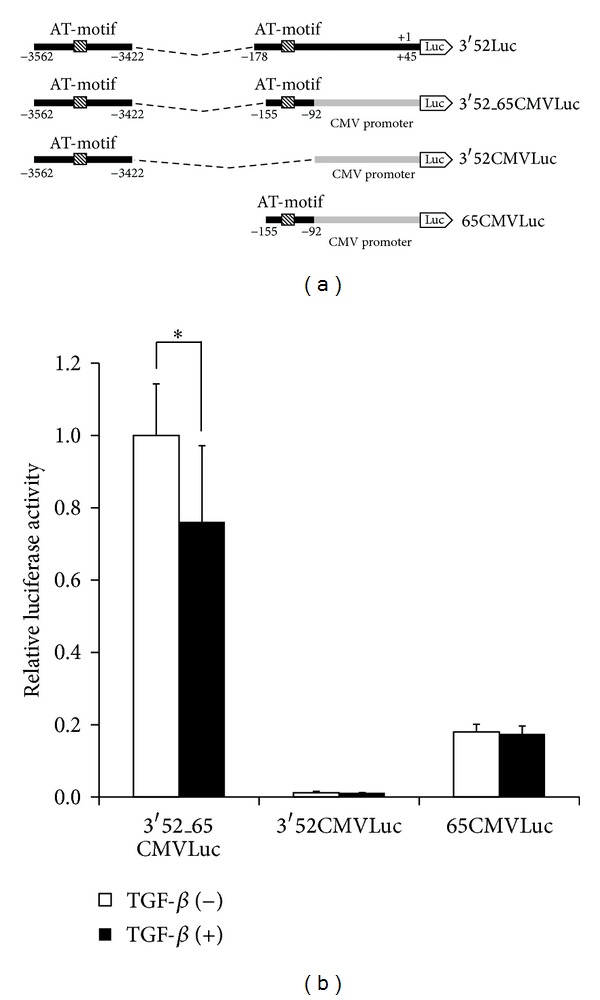
Importance of both distal and proximal AT-motifs for TGF-*β*-mediated repression of the* AFP* promoter. (a) Schematic presentation of CMV Luc conjugated with distal and proximal AT-motifs. (b) HepG2 cells were transfected with each reporter (200 ng) and then stimulated with 5 ng/mL TGF-*β*.

Since there are two functional AT-motifs within the* AFP* promoter between −4995 and +45 (Figure 2(a)), it is possible that the proximal AT-motif also influences TGF-*β*-mediated inhibition of the activity of the* AFP* promoter. To confirm that the proximal AT-motif can also contribute to TGF-*β*-mediated repression of the activity of the* AFP* promoter, the 141nt-length fragment from −3562 to −3422 was ligated to the 64nt-length region between −155 and −92 of the* AFP* promoter. Then, this fragment was further placed at the 5′port of CMV Luc (3′52_65CMVLuc) because the CMV promoter shows the high level of luciferase activity.  Figure 4(b) demonstrates that both the distal and the proximal AT-motifs are needed for TGF-*β* to suppress the activity of the* AFP* promoter.

### 3.5. Association of Smad Proteins with ATBF1

**Figure 5:**
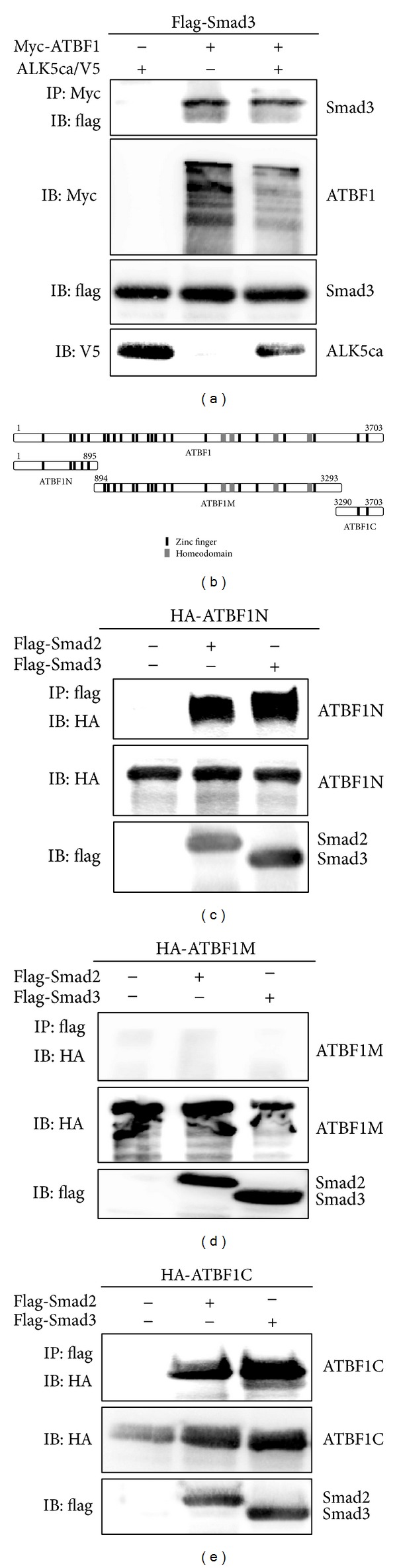
Association of Smads with ATBF1. (a) Interaction of Smad3 with ATBF1. COS7 cells were transfected with Flag-Smad3 (1 *μ*g) with Myc-ATBF1 (2 *μ*g) in combination with ALK5ca/V5 (0.5 *μ*g) or without it. Cell lysates were subjected to immunoprecipitation (IP) with anti-Myc antibody followed by IB with anti-Flag antibody to show the interaction of Flag-Smad3 with Myc-ATBF1. (b) Schematic presentation of ATBF1 mutants. (c–e) Interaction of each ATBF1 mutant with Smad2 or Smad3. COS7 cells were transfected with Flag-Smad2 or Flag-Smad3 (1.5 *μ*g) with HA-ATBF1N (2 *μ*g) (c), HA-ATBF1 M (2 *μ*g) (d), or HA-ATBF1C (2 *μ*g) (e). Cell lysates were subjected to IP with anti-Flag antibody followed by IB with anti-HA antibody to show the interaction of Smad2 and Smad3 with ATBF1 mutants.

**Figure 6:**
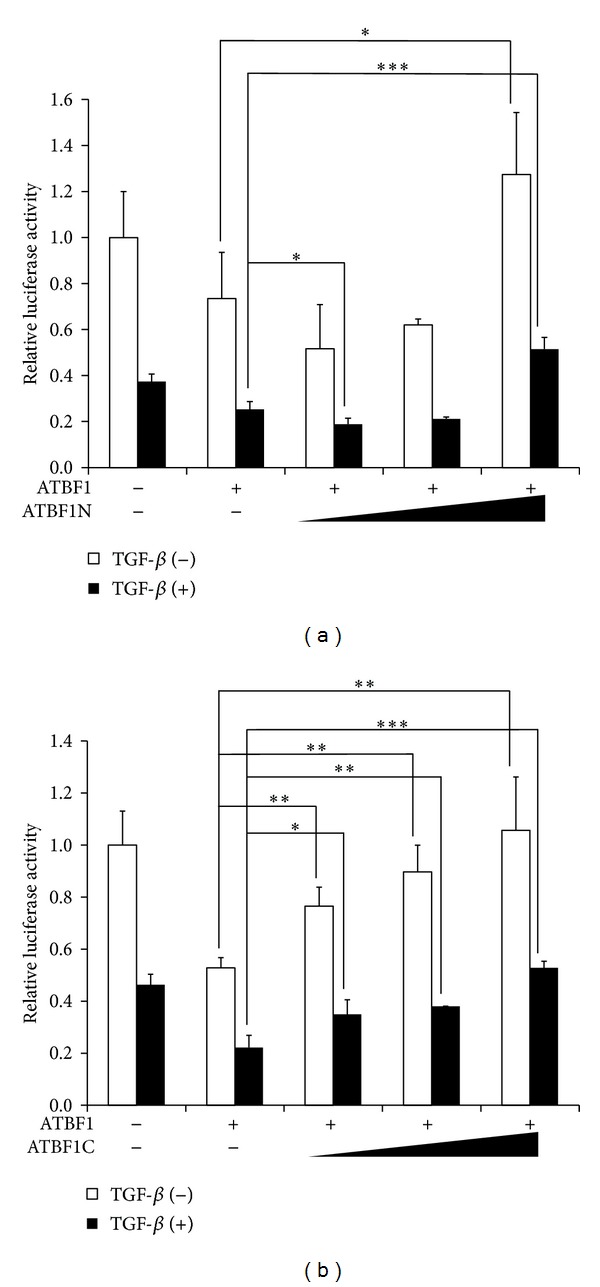
ATBF1N and ATBF1C perturb ATBF1-mediated suppression of the activity of the* AFP *promoter upon TGF-*β* stimulation. ATBF1N (a) and ATNF1C (b) rescue ATBF1-mediated repression of the activity of the* AFP* promoter. HepG2 cells were transfected with increased amounts of either ATBF1N (50, 100, or 200 ng) (a) or ATBF1C (50, 100, or 200 ng) (b) together with ATBF1 (200 ng) and Δ2.7Luc (200 ng). The cells were then stimulated with 5 ng/mL TGF-*β*.

Since ATBF1 cooperates with Smad3 for inhibition of the activity of the* AFP* promoter, we tried to determine whether ATBF1 could interact with R-Smads. Expectedly, ATBF1 could associate with Smad2 and Smad3, although ALK5 activation was not involved in its interaction (Figure 5(a) and data not shown). In these experiments, we could observe multiple bands corresponding to ATBF1. These multiple bands might be due to calpain-1-dependent proteolysis in COS7 cells [35]. To identify the domain(s) of ATBF1 that interact with Smads, we divided ATBF1 into three pieces (Figure 5(b)). ATBF1N as well as ATBF1C had the ability to interact with Smad2 and Smad3, although ATBF1M no longer possessed the ability to bind to either Smad2 or Smad3 (Figures 5(c)–5(e)). It has been reported that DNA-binding domains are present in ATBF1M, including zinc finger domains and homeodomains [12]. When either ATBF1N or ATBF1C was cotransfected with ATBF1, TGF-*β*-mediated inhibition of the activity of the* AFP* promoter was improved owing to their competition with ATBF1 for Smad binding (Figures 6(a) and 6(b)). On the other hand, ATBF1M could inhibit the reporter activity as a dominant negative form of ATBF1 because ATBF1M possesses the ability to bind to AT-motif via its DNA binding domain (data not shown). These results indicate that the N-terminal and C-terminal domains are needed for ATBF1 to interact with R-Smads.

### 3.6. Indirect Binding of Smads to the* AFP* Promoter via ATBF1

**Figure 7:**
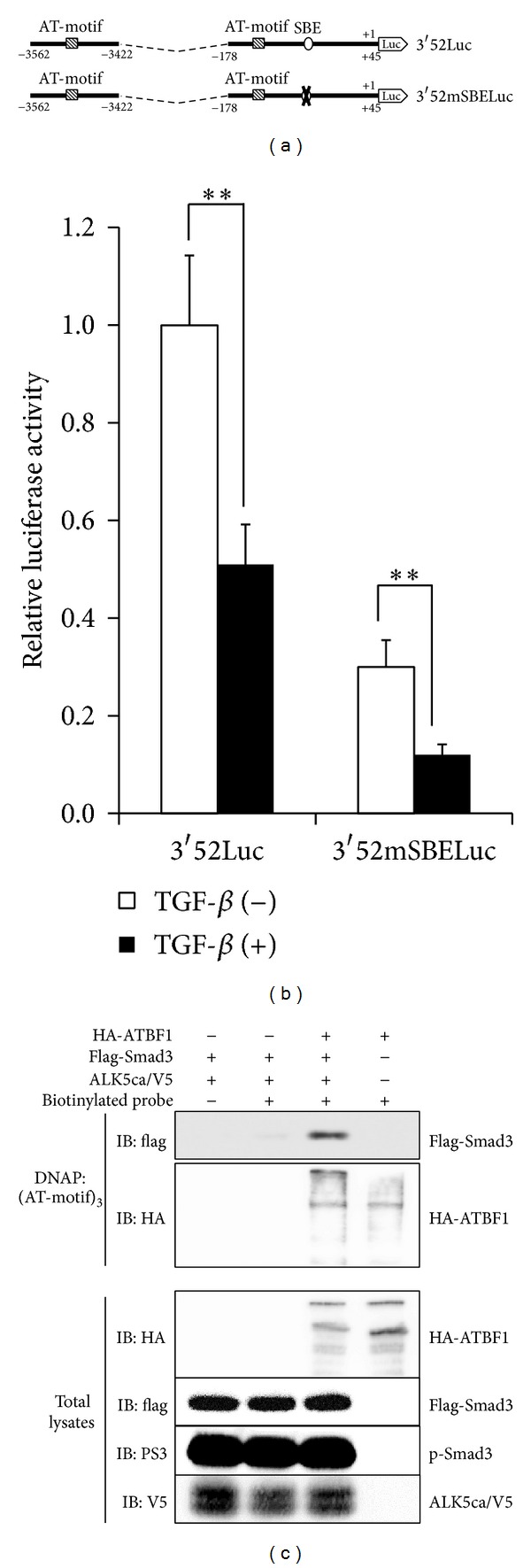
SBE in the proximal region of the* AFP* promoter is not essential for TGF-*β*-mediated suppression of the* AFP *promoter. (a) Schematic presentation of 3′52mSBELuc. A putative SBE, 5′-CAGATA-3′, was mutated to 5′-TTTTTT-3′. (b) Effect of TGF-*β* on 3′52Luc and 3′52mSBELuc activities. HepG2 cells were transfected with each reporter plasmid (200 ng). The cells were then stimulated with 5 ng/mL TGF-*β*. (c) Indirect binding of Smad3 to an AT-motif via ATBF1. The indicated cell lysates were mixed with biotinylated AT-motifs. Then, streptavidin-agarose was added to the mixture. Subsequently, a protein(s) bound to streptavidin-agarose was purified. After SDS-PAGE, IB was carried out using anti-Flag or anti-HA antibody. Using the total lysates, each protein was detected with anti-Flag, anti-HA, anti-V5, or anti-phosphorylated Smad3 (PS3) antibody.

We could find only one Smad binding element (SBE) [36, 37] in 3′52Luc (Figure 7(a)). Thus, we examined whether this SBE is necessary for TGF-*β*-mediated repression of the* AFP* promoter. For that purpose, we made one reporter construct that had mutations within the SBE of 3′52Luc (3′52mSBELuc) (Figure 7(a)). However, 3′52mSBELuc activity was still inhibited by TGF-*β*, although its basal level became weaker than that of 3′52Luc (Figure 7(b)). Thus, the SBE in the* AFP* promoter is not essential for TGF-*β*/Smad signaling to repress the activity of the* AFP* promoter. Indeed, a DNAP assay showed that Smad3 can be detected in the presence of ATBF1 using three repeats of an AT-motif as the probe (Figure 7(c)). Thus, Smad3 probably binds to the promoter region of the* AFP* gene indirectly through its interaction with ATBF1.

## 4. Discussion

**Figure 8:**
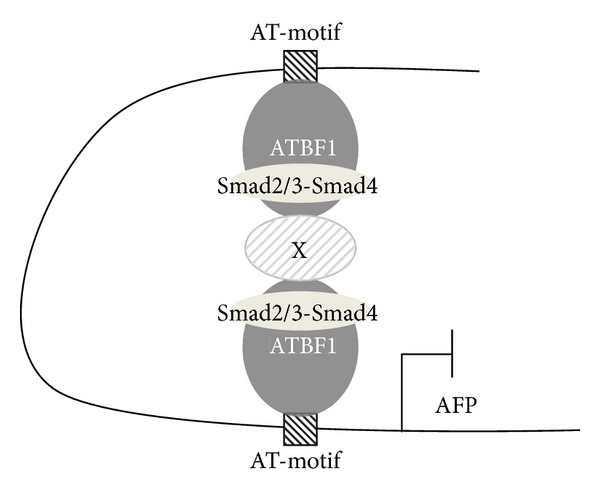
Proposed inhibitory mechanism by which the TGF-*β*/Smad signaling and ATBF1 cooperatively regulate the activity of the* AFP* promoter. The distal and proximal AT-motifs in the* AFP* promoter are bound by ATBF1. Upon TGF-*β* signaling, the R-Smads/Smad4 complex interacts with ATBF1 to make a repressive complex for the activity of the* AFP* promoter. However, whether another protein(s) (termed “X”) is essential for the R-Smad/Smad4/ATBF1 complex remains veiled.

The hepatoma cells HuH-7 and HepG2 cells are known to secrete a large and a low amount of AFP, respectively [38]. A growing body of evidence indicates that the level of AFP in serum from patients is linked to HCC tumorigenicity [3]. p53 has been reported to cooperate with the TGF-*β*/Smad pathway to repress AFP expression via induction of SnoN [22]. However, it remains veiled whether ATBF1, which is known to block* AFP *transcription [4, 10], can also regulate the activity of the* AFP* promoter in consort with the TGF-*β*/Smad pathway because both ATBF1 and TGF-*β*/Smad might possess an antioncogenic function [13–17, 20, 39, 40]. ATBF1 could inhibit the activity of the* AFP* promoter via its regions from −4995 to −2953 and/or from −178 to +45 [4, 26]. Since Δ2.7Luc had a remarkably basal promoter activity compared with 0.2Luc (Figure 2(b)), we initially focused on the distal region of the* AFP* promoter for involvement of the TGF-*β*/Smad signaling with ATBF1. As expected, the TGF-*β*-mediated repressive element in the* AFP* promoter lay near *E*_*B*_, which contains a distal AT-motif. The luciferase reporter conjugated with the inhibitory element including a distal AT-motif (3.52Luc) showed coordinated inhibitory action between the TGF-*β* signaling and ATBF1 (Figure 3(f)). Therefore, we were struck with the notion that ATBF1 physically interacts with Smads, which play key roles in the canonical TGF-*β* signaling pathway [18–20]. Not surprisingly, ATBF1 could interact with Smad2 and Smad3, although C-terminal phosphorylation of R-Smads was dispensable (Figure 5(a) and data not shown). In addition, both the N-terminal (ATBF1N) and the C-terminal mutants of ATBF1 (ATBF1C), but not the middle domain of ATBF1 (ATBF1M) which possesses DNA-binding activity [12], could interact with R-Smads (Figures 5(c)–5(e)). We hypothesized that both ATBF1N and ATBF1C might compete with wildtype ATBF1 for Smad binding to rescue ATBF1-mediated repression of the* AFP* promoter upon TGF-*β* stimulation because both of them have the ability to bind to R-Smads. According to our expectation, both ATBF1N and ATBF1C improved the reporter activity decreased by the cooperative ATBF1 and TGF-*β* signaling (Figures 6(a) and 6(b)). As shown in Figure 2(b), 0.2Luc activity could also be reduced upon TGF-*β* stimulation despite very low basal promoter activity. Because of the presence of a proximal AT-motif within this region, we speculated that a proximal AT-motif also contributes to TGF-*β*-mediated-repression of the activity of the* AFP* promoter. The deletion of a proximal AT-motif from 3′52_65CMVLuc led us to recognize the importance of a proximal AT-motif as well as a distal AT-motif for TGF-*β*-mediated repression of the activity of the* AFP* promoter (Figure 4(b)). A Smad binding element (SBE) composed of consensus 5′-AGAC-3′ is flanked to a proximal AT-motif. However, the mutation of the SBE in 3′52Luc showed reduced basal promoter activity but still maintained TGF-*β*-mediated repression of the* AFP* promoter (Figure 7(b)). Thus, Smad might not be necessary to bind to a promoter element(s) in the* AFP* gene directly when ATBF1 cooperates with TGF-*β* signaling, whereas p53-mediated repression of the* AFP* promoter activity needs direct binding of Smads to DNA [22, 23]. The requirement of direct DNA binding for R-Smads is probably dependent on which transcriptional regulator(s) they cooperate or synergize with. Indeed, Smad3 could bind to AT-motifs in the presence of ATBF1, but not in its absence (Figure 7(c)). Although we do not know if the ATBF1/Smad complex at a distal AT-motif can physically interact with that at a proximal AT-motif directly or indirectly through another protein(s) (“X”; Figure 8), both AT-motifs are definitely important for cooperation between ATBF1 and the TGF-*β* signaling to suppress the activity of the* AFP* promoter.

ATBF-1 is known to compete with HNF-1 for binding to AT-motifs in the AFP promoter [4]. Thus, the following possibility can be speculated; upon TGF-*β* stimulation, Smads can not only bind to AT-motifs via ATBF-1 indirectly but also interact with HNF-1 to dissociate it from AT-motifs. Due to dual function of Smads, the* AFP* prompter activity might be inhibited. This possibility is interesting to be investigated.

p53-mediated repression of the* AFP* promoter activity needs direct binding of Smads to SBE/p53RE. Wilkinson et al. further demonstrated that mSin3A and SnoN which is a direct target gene of TGF-*β* signal are recruited by p53/Smads complex when the transcription of the* AFP* gene is inhibited [22, 23]. In our current study, the region including SBE/p53RE in the* AFP* promoter was not investigated because the basal reporter activities of 3Luc, 1.6Luc, and 0.9Luc were very low compared with that of Δ2.7Luc (Figure 2(b)). It is possible that ATBF1 might interplay with p53 via Smad complex to effectively repress the transcription of the* AFP* gene. Further experiments are needed in the future to confirm these hypotheses as well.

## 5. Conclusion

In summary, we report a novel interaction between ATBF1 and Smads. The interaction between ATBF1 and Smads appears to cooperatively inhibit the transcription of* AFP* gene upon TGF-*β* signaling. In particular, both proximal and distal AT-motifs are required for TGF-*β*/Smad signaling to counteract with the transcription of the* AFP* gene.

## Figures and Tables

**Table 1:** PCR primers to amplify human cDNAs.

Human cDNA		Sequence	*L*	AT	Cycle
AFP	(+)	ATCCAGGAGAGCCAAGCATT	434	58°C	20
	(−)	TTCATCGTTTGCAGCGCTAC			

ATBF1	(+)	TGGCATCAAGTACAGCGCTC	392	53°C	35
	(−)	GAACAGTTGTGCTGGGCAGA			

TMEPAI	(+)	GATCATCATCATCGTGGTGG	455	60°C	35
	(−)	CACTGTCGAAGATGGTTCTG			

*β*-actin	(+)	CACCCACACTGTGCCCATCTACGA	410	63°C	22
	(−)	TGGCGTACAGGTCTTTGCGGATGT			

*L*: length of PCR fragment; AT: annealing temperature.
